# Mechanical Properties of Cellulose Aerogel Composites with and without Crude Oil Filling

**DOI:** 10.3390/gels10020135

**Published:** 2024-02-07

**Authors:** Tatjana Paulauskiene, Egle Sirtaute, Arturas Tadzijevas, Jochen Uebe

**Affiliations:** 1 Engineering Department, Faculty of Marine Technology and Natural Sciences, Klaipeda University, H. Manto 84, 92294 Klaipeda, Lithuania; egle.sirtaute@ku.lt (E.S.); jochen.uebe@ku.lt (J.U.); 2 Marine Research Institute, Klaipeda University, H. Manto 84, 92294 Klaipeda, Lithuania; arturas.tadzijevas@ku.lt

**Keywords:** aerogel, sorbent, sorption, oil spill, paper-waste, composite, waste valorization, fiber hemp

## Abstract

Aerogels are an excellent alternative to traditional oil absorbents and are designed to remove oil or organic solvents from water. Cellulose-based aerogels can be distinguished as polymers that are non-toxic, environmentally friendly, and biodegradable. The compression measurement properties of aerogels are often evaluated using dry samples. Here, oil-soaked, hydrophobized cellulose aerogel samples were examined in comparison to dry samples with and without additional hemp fibers and various levels of starch for crosslinking. The samples were characterized by compression measurement properties and filmed to evaluate the regeneration of the sorbent with repeated use. Overall, the measurements of the mechanical properties for the dry samples showed good reproducibility. The Young’s modulus of samples with additional hemp fibers is significantly increased and also shows higher strength than samples without hemp fibers. However, samples without hemp fibers showed slightly better relaxation after compression. Oil acts as a weak plasticizer for all aerogel samples. However, it is important to note that the oil does not cause the samples to decompose in the way unmodified cellulose aerogels do in water. Therefore, using hydrophobized cellulose aerogels as sorbents for oil in a sea or harbor with swell means that they can be collected in their entirety even after use.

## 1. Introduction

Aerogels are an excellent alternative to traditional oil absorbents and are designed to remove oil or organic solvents from water. Research on aerogels began more than 90 years ago, but the greatest activity has occurred in recent decades, when aerogels have been used in the construction sector, for special thermal or sound insulation, in the water or air purification sector, in the automotive sector in the production of electric car batteries, and in the space sector. Taking into account the latest trends, ecological ways to purify water pollutants are being sought and for this purpose aerogels made from natural materials are used—mostly cellulose-based aerogels [1,2]. An overview of cellulose and its derivatives in aerogels form is provided in [3]. Given its status as the most abundant natural polymer on Earth and its prevalence as the primary component of the second most common type of waste—paper and cardboard [4], cellulose emerges as the preferred raw material for producing aerogels in this study. Cellulose-based aerogels can be distinguished as polymers that are non-toxic, environmentally friendly, and biodegradable. Aerogels modified with methyltrimethoxysilane (MTMS) are hydrophobic, to improve the specificity for oil. Despite the excellent properties of aerogels, which include high porosity, low density, and high surface area [5], the mechanical properties of cellulose-based aerogels still need to be improved for use at high temperatures or organic solvent removal [6]. Little literature is available about the reuse of aerogels close to real-world conditions, namely the purification of oil from water and the multiple use of aerogels [7,8,9]. Currently, the mechanical properties of aerogels are evaluated using dry samples and only one-time compression [1,10,11].

A recent article mentions cyclic compression testing of nanofibrillated aerogels to evaluate the durability of samples after 50 compression-unloading cycles, but also uses non-oil-sorbed aerogels [12]. There are almost no studies attempting cyclic mechanical testing with oil or organic liquids, and there are a few studies in which aerogel is soaked in oil and mechanically squeezed in ethanol [13] to evaluate shape relaxation [6].

In this paper, the mechanical properties of dry and oil-soaked natural cellulose-based aerogels were investigated to evaluate their applicability in the field of water treatment. For this purpose, cellulose aerogels made from paper-waste, starch as a crosslinker, and agriculture residue (hemp straw fibers) were used, whose preparation and oil sorption capacity were already published by the authors of this paper in [14]. Young’s modulus and initial height recovery of the aerogel samples were determined and evaluated.

## 2. Results and Discussion

First, the results and discussion of the dry aerogel samples are presented below, which serve as a reference for the oil-sorbed samples.

The stress–strain curves of both MTMS-modified and unmodified cellulose aerogels corresponded to the three different deformation ranges, which [2] has also described. Similar behavior was observed for the morphology of the porous foam type [15,16].

**Figure 1:**
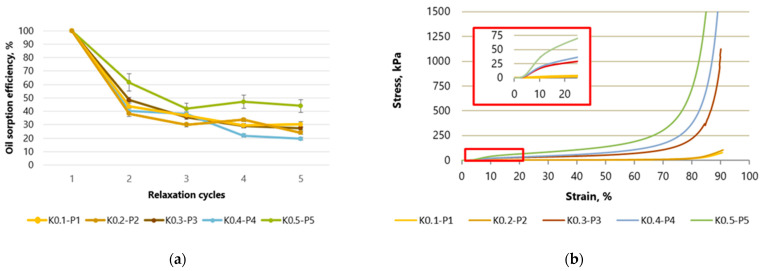
Compressive stress and strain curves for samples with starch as a binder: (**a**) variation of aerogel efficiency during compression–regeneration cycles; (**b**) maximum aerogel deformation up to 90%. K—starch, P—paper-waste.

**Figure 2:**
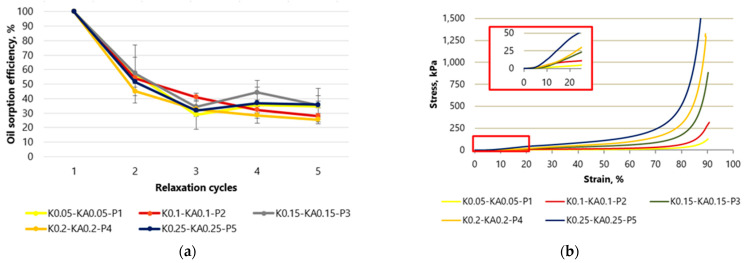
Compressive stress and strain curves for samples with starch and fiber hemp as binders: (**a**) variation of aerogel efficiency during compression–regeneration cycles; (**b**) maximum aerogel deformation up to 90%. K—starch, KA—hemp, P—paper-waste.

In Figure 1 and Figure 2 of the samples tested at 10 different contents of starch or hemp fibers, a clear linear range of stress and strain can be seen. The number next to the letter K indicates the amount of starch in grams of the samples, KA is the amount of hemp in grams of the sample, and P is the amount of cardboard. In cellulose-based samples in which only starch is used as a binder, the linear range can be seen to extend from 5 to 10% strain (Figure 1b), and in samples in which starch is used with hemp fibers, this area deforms slightly further up to 20% (Figure 2b).

Figure 1a shows the sorption capacity in % based on the first value of the control samples made of cellulose fibers with starch without hemp fibers. The absolute sorption capacity in g g^−1^ has been published in Figure 5 in [14]. At a compression of 90%, the sorption capacity of crude oil drops significantly by up to 43% after the first cycle. After the second cycle, the sorption capacity fell to 30% and remained at this level in subsequent cycles. For the samples with the highest concentration of cellulose and starch, K0.5-P5, the drop in sorption capacity was lower, presumably due to their higher density and lower porosity. The maximum crude oil sorption capacity of these samples drops by 61% after the first compression and reaches 44% initial efficiency after the fifth compression. The reason for the large reduction in absorption capacity after the first cycle is the massive structural collapse after the first compression, which causes the pores to collapse as an oil absorption medium [17].

By evaluating the data shown in Figure 2a, it can be seen that the aerogel samples containing hemp fiber showed similar results to the control samples. After the second cycle, a decrease in sorption efficiency was observed for samples of all cellulose concentrations. The sorption capacity of the sample with the lowest K0.05-KA0.05-P1 cellulose concentration was 57% after the second compression cycle and reached 34% after all five cycles. Meanwhile, the sorption capacity of the K0.25-KA0.25-P5 aerogels with the highest concentration (K0.25-KA0.25-P5) was 51 after the second cycle and reached 35% after all cycles. A possible explanation for the higher sorption capacity observed in the cellulose aerogel samples containing hemp fibers during the second compression-regeneration cycle, compared to cellulose aerogels without hemp fibers, could be attributed to the nature of hemp fibers derived from agricultural residue. These fibers are not delignified and thus maintain greater stiffness compared to cellulose sourced from heavily processed paper. Consequently, cellulose aerogels incorporating hemp fibers may exhibit enhanced mechanical resilience, enabling them to better withstand the applied mechanical stress. During the tests, the aerogel returning to its original position easily sorbed the oil on the foil plate, causing weight inaccuracy.

When aerogels are compressed as shown in Figure 1 and Figure 2, the cross-sectional area does not change in most samples, and only a few samples collapse. This can be observed both when using starch as an additive and when combining starch with fiber hemp. In study [18], the linear elastic region is preceded by a contact region in which the stresses due to the surface roughness of the sample are low until the clamps of the testing machine come into contact with the material. In the curves of the studies carried out, it can be seen that the contact area for the samples with starch as an additive and starch with fiber hemp extends to about 5% compression, from which point the range of linear elasticity begins.

The middle range (10–20%) of slowly increasing stresses of the dry cellulose aerogel samples with and without hemp fibers correlates with the descriptions of the curve shown by researchers [19]. This region of the compression curve has a small slope, which means that porous materials do not experience a large increase in stress upon further deformation. According to [20], we see progressive elastic buckling of the fiber walls, followed by a region of pure material compression where the fiber walls are in contact with each other. The irreversible loss of compressive strength and stress is accompanied by the irreversible loss of porous structure, thereby reducing the porosity of the samples. The broken fiber is compressed and gradually deforms further until eventually the material itself begins to compress. At the end of the compression experiments, up to 90% can no longer regain their original shape and height.

This third range (60–90%) is characterized by a sharp increase in the slope of the curve, which means that the material is being compacted. This range starts between 60 and 70% of the strain for both groups of samples. In general, an increase in cellulose concentration, which leads to an increase in the density of aerogels, leads to a decrease in pore size and an increase in fiber wall thickness. This increases the resistance to bending or crushing of the fiber walls, resulting in higher elastic moduli and stresses in the middle region of the stress–strain curve, and the fiber walls come into contact with each other more quickly, resulting in a reduction in the stress at which compaction begins.

Compared to synthetic polymer aerogels, the studied cellulose-based aerogels with or without fiber hemp did not show a clear horizontal region of plastic deformation at low strains. Instead, these aerogels showed a gradual transition into the pure material compression regime above the 10% deformation limit for control samples and the 20% deformation limit for samples with hemp fibers.

The elastic modulus, apparent yield strength, and compressive strength of all samples were improved with increasing fiber content. According to study [21], increasing the amount of aerogel fibers reduces the ratio of large to fine pores in the structure and increases the overall bulk density of the aerogel. Therefore, as the load increases, the flatter structures made of irregular cellulose fibers begin to come into contact with each other and act as load-bearing zones, thereby improving the mechanical properties of the aerogel. Aerogels with higher cellulose density are characterized by strong hydrogen bonds and sufficient physical entanglement, which has a positive effect on the flexibility and elasticity of aerogels. However, too high a density leads to irreversible compaction and loss of elastic properties, making aerogels brittle [21].

In the next section, the results of the compressions of the oil-sorbed aerogel samples are presented.

### 2.1. Studies on the Regeneration and Sorption of Aerogels

It was found that the samples regained some of their original height after the first compression cycle, while the shape relaxation was negligible from the second to the fifth cycle. This is explained by the fact that aerogels with higher concentration have a denser structure and fewer pores, which means that the compression stage of the pure material is reached more quickly. Such deformation is irreversible and the stress–strain curves increase exponentially. Therefore, the oil-soaked samples were only pressed up to 70% strain to prevent collapse.

**Figure 3:**
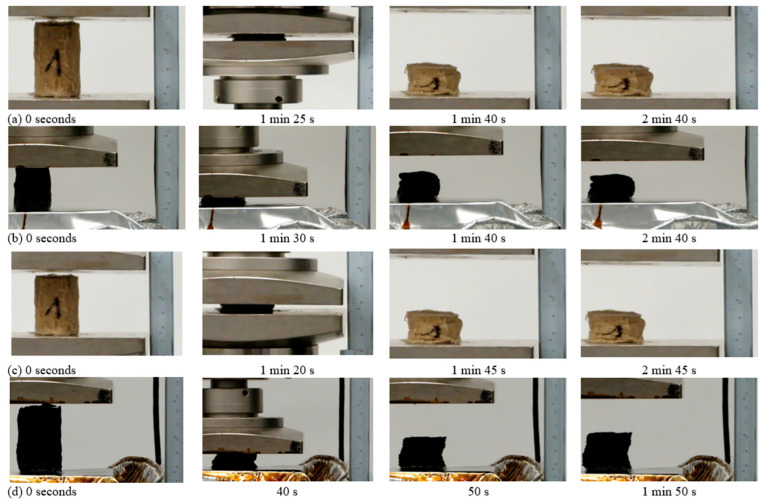
Investigations of the compressive mechanical properties of cellulose aerogels: (**a**) deformation of samples up to 90% of their deformation limits when testing dry samples from paper-waste and starch K0.1-P1; (**b**) deformation–relaxation tests up to 70% deformation limits of samples based on samples made from paper-waste and starch K0.1-P1, or (**c**,**d**) by inserting the hemp fiber K0.05-KA0.05-K1.

Figure 3 shows what the dry samples of the lowest cellulose aerogel samples K0.1-P1 and K0.05-KA0.05-P1 look like during compression testing. Samples with the lowest cellulose and starch content, K0.1-P1, relax 42% of their original height after 90% deformation after 2 min 40 s. For the samples with fiber hemp such as K0.05-KA0.05-P1, the initial height improvement was 44%.

The same figure also shows oil-sorbed samples that were compressed up to 70%. Samples with the lowest content of starch K0.1-P1 even relaxed 56% of their original height (see 2 min 40 s in Figure 3). The same relaxation is also achieved with samples that have the lowest content of the mixture of starch and fiber hemp (K0.05-KA0.05-P).

**Figure 4:**
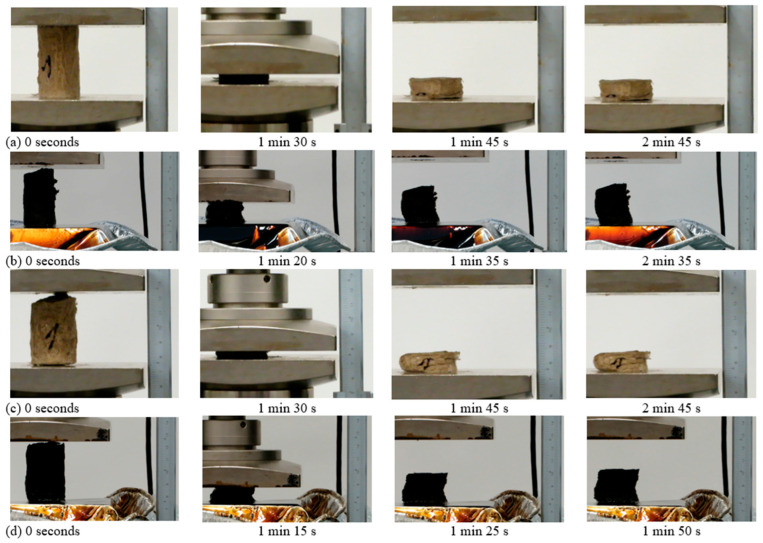
Investigations of the compressive mechanical properties of cellulose aerogels: (**a**) deformation of samples up to 90% of their deformation limits when testing dry samples of paper-waste and starch K0.5-P5, first cycle; (**b**) deformation–relaxation up to 70% deformation limits of samples based on samples made from paper-waste and starch K0.5-P5, or (**c**,**d**) by inserting the hemp fiber K0.25-KA0.25-K5.

Figure 4 shows the results of testing aerogel samples with the highest cellulose concentration. K0.5-P5 concentration samples (see 2 min 45 s in Figure 4) relaxed by 27%, while the samples with hemp fibers (K0.25-KA0.25-P5) relaxed slightly more at 30% after 2 min 45 s. Oil-sorbed samples with starch (K0.5-5) after 2 min 35 s relaxed more strongly, namely by 61%, compared to the comparison samples, but at approximately the same speed. The same applies to the sample with starch and hemp fibers, which, however, is faster than the dry comparison sample (1 min 50–53 s).

In summary, oil-sorbed samples showed faster and more extensive relaxation than dry samples. The relaxation of the oil-sorbed samples with the lowest concentrations (K0.1-P1 and K0.05-KA0.05-P) was 56% of the original height, and of the samples with the highest concentrations (K0.5-P5 and K0.25-KA0.25-P5), the control samples with starch showed the best results—they relax around 61%. An explanation for this result could be similar to [22], according to which aerogel made from cellulose provides improved mechanical strength after adding another polysaccharide.

There are relatively few articles with petroleum-sorbed cellulose aerogels; the aerogels are usually silica-based. A recent study by Wu et al. states that for cellulose aerogels, the final shape relaxation is 70%. The deformation reached 80% after the first cycle and less than 40% after the next cycles [23].

The compressive stiffness of aerogel composites generally depends on the ability of the microstructure to effectively transmit the applied stress and also depends on the amount of additives used, which increase the density of the aerogels and make the composite stiffer [24,25].

Based on these results, it would be possible in the future to determine the force distribution in the material using simulation programs. Such research is currently being carried out, for example, on silicon-based [26,27] and graphene aerogels [28,29].

**Figure 5:**
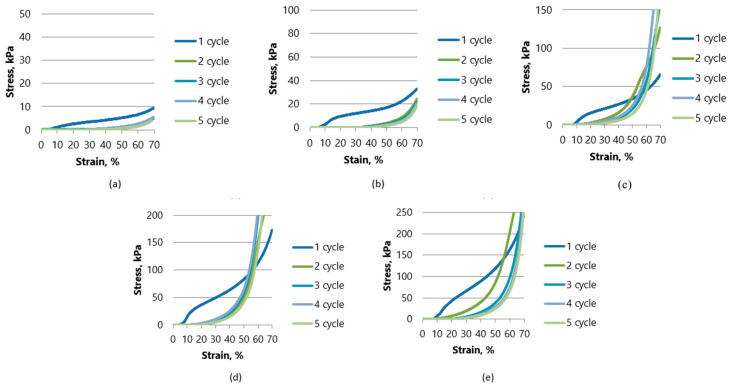
Compressive stress and deformation curves for 5 cycles up to 70% deformation for samples with starch (K) as binder: (**a**) K0.1-P1; (**b**) K0.2-P2; (**c**) K0.3-P3; (**d**) K0.4-P4; (**e**) K0.5-P5.

**Figure 6:**
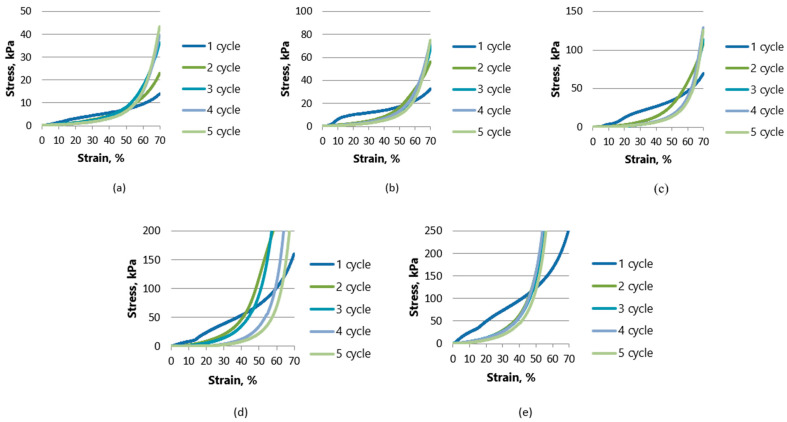
Compressive stress and deformation curves for 5 cycles up to 70% deformation test for samples with starch (K) and fiber hemp (KA) as binders: (**a**) K0.05-KA0.05-P1; (**b**) K0.1-KA0.1-P2; (**c**) K0.15-KA0.15-P3; (**d**) K0.2-KA0.2-P4; (**e**) K0.25-KA0.25-P5.

To evaluate the influence of different additives on the mechanical properties, a mechanical measurement was carried out on control samples and samples with hemp fibers. A typical stress–strain curve is shown in Figure 5 and Figure 6. Visually, none of the samples lost their original shape, and they did not crack and did not break during the test; both types of aerogels showed plastic behavior. The graphics show the behavior of aerogels in five cycles. In the first few cycles, a region of linear deformation, an intermediate compression zone, and an exponentially increasing curve indicating the compression of the pure material are clearly visible. Aerogels with lower cellulose concentrations show more plastic behavior after the first cycle and the curves for cycles 2–5 move closer to the X axis. Meanwhile, the stress–strain curves begin to increase exponentially with an increasing density of the aerogel (at 4 and 5% of the amount of additive used) as the compression range of the pure material is reached more quickly.

The strength of the oil-sorbed sample K0.1-P1 reaches 9.3 kPa. Meanwhile, the strength of the K0.5-P5 aerogel with the highest cellulose concentration reached almost 250 kPa. When comparing the oil-sorbed samples in which starch was used with fiber hemp as an additive, the strength of the sample K0.05-KA0.05-P1 reached 14 kPa, while the strength of the samples with the highest concentration (K0.25-KA0.25-P5) reached almost 256.5 kPa. It can be concluded that the lowest-density aerogels when starch and hemp are used as additives had 50.5% higher strength than the starch-only samples, while the aerogels with the highest cellulose concentrations were quite similar in strength—250 kPa—K0.5-P5 and 256.5 kPa—K0.25-KA0.25-P5.

From Figure 3 and Figure 4 described previously, it can also be seen that the relaxation was better for the oil-sorbed samples.

### 2.2. Determinations of the Young Modulus

After conducting compression tests on dry samples up to 90% deformation using starch as additives and starch with fiber hemp, we can see that samples up to the second concentration of K0.2-P2 had a lower numerical value of Young’s modulus after conducting compression tests on dry samples up to 90% deformation using starch as additives and starch with fiber hemp. However, from the third concentration, specifically K0.3 -P3, an even lower value was observed (Table 1), beyond which the third concentration, however, of K0.3-P3 had an even lower one (Table 1).

The compressive Young’s modulus (E) calculated during measurements is the slope of the linear part of the stress–strain curve in the elastic region. It is equal to the stress change (Δσ) divided by the strain change (Δε).

The numerical value of Young’s modulus was demonstrated by samples of cellulose waste, fiber hemp, and starch (K0.15-KA0.15-P3; K0.2-KA0.2-P4; K0.25-KA0.25-P5). The compressive stress–strain curves show considerable reasonable flexibility of the aerogels.

In the compression tests on oil-sorbed samples without hemp fibers with a compression of up to 70% and samples with hemp fibers as additives, it can be seen that after a single use and compression of the samples after the first cycle, the pore structure breaks and does not relax (Table 2).

For oil-sorbed samples, the numerical value of the Young’s modulus is lower for samples with a higher proportion of starch (K0.2-P2). From the samples K0.3-P3 onwards, a similar trend can be seen as with the previously tested dry samples—the numerical values of the Young’s modulus become higher for samples with starch and fiber hemp. For samples with the lowest concentration (K0.1-P1), the numerical value of the Young’s modulus (calculated Young’s modulus value—E) reaches 3.461 kPa; for samples with the highest concentration (K0.5-P5), it reaches 1291.095 kPa. On the other hand, when starch and fiber hemp are used as additives, the numerical value of Young’s modulus of the lowest concentration (K0.05-KA0.05-P1) reaches 22.402 kPa, and for the samples with the highest concentration (K0.25-KA0, 25-P5), it reaches 320.269 kPa.

It can be mentioned that sugarcane-based aerogels had a numerical value of the Young’s modulus of 88 kPa [30]; for rice-based aerogels, the numerical values of the Young’s modulus were between 2.5 and 15.6, depending on the density kPa [31], and rice-straw-based aerogels had the highest numerical value of Young’s modulus of 47 kPa [32].

In Figure 6a, the tendency can be observed that the Young’s modulus also increases as the increasing density of the sample. The material becomes stiffer, but has fewer pores and absorbs oil and its products more poorly. This trend increase is observed for both dry and oil-sorbed samples. At the lowest amount of cellulose in the samples, when starch (K0.1-P1) was used as an additive, the density of the sample reached 0.017 g cm^−3^ and the numerical value of Young’s modulus was 4 kPa. In samples with the highest cellulose concentration using starch (K0.5-P5) as an additive, the density of the sample was 0.076 g cm^−3^, and the numerical value of Young’s modulus was 937 kPa.

**Figure 7:**
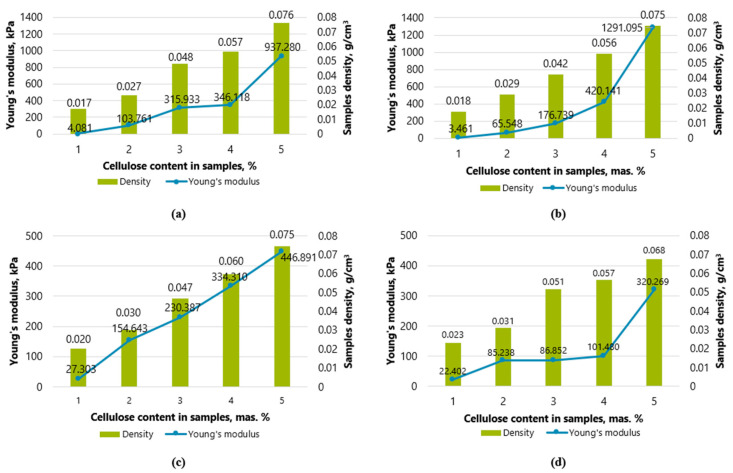
Dependence of Young’s modulus on the aerogel density: (**a**) dry and (**b**) wet samples with starch as an additive, and (**c**) dry and (**d**) oil-sorbed samples with starch and fiber hemp as an additive.

In Figure 7a, it can be seen that as the density of the sample increases, the numerical value of the Young’s modulus also increases. This trend increase can be observed for both dry and oil-sorbed samples. Article [33] describes the Young’s modulus of pineapple-leaf-based aerogels (PF). The Young modulus increases as the amount of pineapple leaf increases from 0.5 to 1 and 2% by weight. The PF concentration strengthened the aerogel structure, and PF aerogels had a low numerical value of Young’s modulus (0.47–7.86 kPa) compared to other cellulose aerogels (13–39 kPa). In this work, the density of the aerogel sample at the lowest amount of cellulose in the samples when starch (K0.1-P1) was used as an additive reached 0.017 g cm^−3^ and the numerical value of Young’s modulus was 4 kPa. In samples with the highest cellulose concentration using starch (K0.5-P5) as an additive, the density of the sample was 0.076 g cm^−3^, and the numerical value of Young’s modulus was 937 kPa. This means that the prepared aerogels showed good elasticity.

In Figure 7c, you can see the aerogel samples in which fiber hemp and starch were used as additives. At the lowest amount of cellulose in the samples (K0.05-KA0.05-P1), the density reached 0.020 g cm^−3^ and the numerical value of Young’s modulus was 27 kPa. In samples with the highest cellulose concentration using starch (K0.25-KA0.25-P5) as an additive, the density of the sample was 0.075 g cm^−3^, and the numerical value of Young’s modulus was 447 kPa. It can be seen that up to the second cellulose concentration, the numerical values of the elastic modulus of the oil-sorbed samples (K0.1-KA0.1-P2) were higher than those of the dry samples, and from K0.15-KA0.15-P3, the numerical values of the elastic modulus were lower than dry samples. Therefore, only samples with a lower cellulose concentration can be considered to have better elasticity when using starch and fiber hemp as additives, indicating that the material is easy to stretch and deform.

Looking at the data of the oil-sorbed samples shown in Figure 7b, we notice that at the lowest amount of cellulose in the samples, when starch (K0.1-P1) was used as an additive, the density of the sample reached 0.018 g cm^−3^ and the numerical value of Young’s modulus was 3 kPa. In samples with the highest cellulose concentration using starch (K0.5-P5) as an additive, the density of the sample was 0.075 g cm^−3^ and the numerical value of Young’s modulus reached 1.3 MPa.

When comparing the data in Figure 7b,d, the Young’s modulus of the you can see that for oil-sorbed samples with starch and hemp fiber hemp up to the second cellulose concentration (K0.15-KA0.15-P3), the numerical values of the Young’s modulus are higher than those in Figure 7b of samples without hemp fibers regarding the strength of the numerical values of the Young’s modulus. However, samples K0.3-P3, K0.4-P4 and K0.5-P5 show higher Young’s moduli, namely 117 kPa, 420 kPa and 1.3 MPa, respectively, exceeding the calculated numerical values of Young’s modulus of 87 kPa, 101 kPa and 320 kPa for the samples with hemp. However, with increasing concentration, concentration samples K0.3-P3, K0.4-P4, and K0.5-P5 show better results, whose numerical values of Young’s modulus reach 117 kPa, 420 kPa, and 1.3 MPa, which belong to the samples with starch and exceed the numerical values of Young’s modulus calculated for hemp—87 kPa, 101 kPa, and 320 kPa, respectively.

The authors of [34] investigated modified kapok/cellulose (KNA) and kapok/microfibrillated cellulose (KMA) aerogels. The article mentions that the amount of kapok fiber exceeds 0.3% by weight, and the mechanical properties of aerogels deteriorated due to structural instability. A similar trend can be seen in this work, where hemp fiber content increases, and the mechanical properties of the samples become worse compared to for samples containing only starch.

## 3. Conclusions

Overall, the measurements of the mechanical properties for the dry samples show good reproducibility, although this decreases somewhat for the oil-sorbed samples due to the increased moisture. After wetting the aerogel, its structure becomes close to hydrogel and the pore structure loses its properties. The samples with different concentrations of starch and hemp fibers reveal a clearly linear range of stress and strain. For dry cellulose-based samples using starch as a binder, it can be seen that the linear range extends from 5 to 10% deformations, and for dry samples using starch and hemp fiber as a binder, this range extends slightly further—from 5 to 20% deformations.

The Young’s modulus of samples with additional hemp fibers is significantly increased and also shows higher strength than samples without hemp fibers. However, samples without hemp fibers showed slightly better relaxation after compression.

Oil acts as a weak plasticizer for all aerogel samples and slightly reduces the strength of the samples as well as the extent of relaxation after compression. However, it is important to note that the oil does not cause the samples to decompose in the way unmodified cellulose aerogels do in water. A low numerical value of Young’s modulus would help aerogels not lose their original shape, break, and crumble under waves. Therefore, it can be expected that when hydrophobized cellulose aerogels are used as sorbents for oil in a sea or harbor with swell, they can be collected in their entirety even after use.

## 4. Materials and Methods

### 4.1. Materials

The production of the cellulose aerogel samples used in this study from cardboard paper-waste and starch and partly with and without hemp fibers is described in [14]. Information about the structure and morphology of the cellulose aerogels can be found in [35]. A total of 250 samples of different compositions were produced, so every measurement was repeated 5 times. The dimension of the samples containing only starch ranged from 3.8 to 5.1 cm in height and 2.8 to 2.9 cm in diameter, while samples with starch and hemp fiber ranged from 4 to 4.4 cm in height and 2.7 to 2.9 in diameter. The composition of the samples without hemp fibers is shown in Table 3.

Table 4 lists the composition of the aerogel samples with additional hemp fibers.

The samples were prepared as described in [14], and they were hydrophobized in order to prepare them for oil sorption. The Characterization as a sorbent for oil involved analyzing the porosity, density, contact angle of the samples against water, and the sorption capacity, as outlined in [14], following common practices in the literature [3]. To characterize them as a sorbent for oil, analyzing the porosity, the density, the contact angle of the samples against water, and the sorption capacity [2] as stated in study [32] is common.

Results of the density and porosity of aerogels are presented in Table 5 and Table 6.

Crude oil with a density of 867 kg m^−3^ and a dynamic viscosity of 0.0097 Pa s from SC ORLEN Lietuva (Mazeikiai, Lithuania) was used.

### 4.2. Investigations of Compressive Mechanical Properties—Maximum Deformation of the Samples

The Zwick/Roell Z020 testing machine was utilized for the tests. The force cell was calibrated to 0.1 kN with a 1% error margin. The maximum force recorded during the test was 240 N at the conclusion of compression. The elastic phase of compression for aerogels concluded at approximately 0.5 to 1.5 N. To enhance the accuracy of force cell data readings, a time filter with a 100 ms averaging function was applied.

During the tests, standard compression grips designed for the Zwick/Roell Z020 machine were employed. The lower grip remained fixed in all directions, while the upper grip was secured with a standard ball joint to guarantee parallel compression.

A Zwick/Roell Z020 universal testing machine with the TesteExpert II program (Zwick/Roell, Ulm, Germany) with the strain rate of 1 mm min^−1^ was used for the investigation. Before carrying out compression tests, the geometric dimensions of the samples—diameter and height—were determined.

First, the maximum deformation was determined on dry samples in order to compress up to 90%. It was observed that oil-sorbed samples compressed up to 90% did not regain their initial height; therefore, 70% sample deformation was chosen.

The procedure for determining the mechanical properties of oil-sorbed samples was as follows: during the study, 5 cycle tests were carried out with the same sorption material up to 70% compression. First, the aerogel sample was immersed in oil for 2 min and then placed on a perforated mesh for 1 min to remove excess liquid from the sample surface. Then, the sample was weighed and placed between the clamps of the Zwick/Roell Z020 universal testing machine, where it was pressed with constant force up to 70% compression. The entire process was filmed to evaluate the regeneration of the sorbent with repeated use. After compression, the sample was allowed to relax for 1 min. The process was then repeated for each further compression cycle.

The elastic modulus was calculated using the secant method to mitigate the nonlinearity of the compression curve beginnings. Curve readings were taken at 3% and 5% of relative linear elongation.

## Figures and Tables

**Table 1:** Compression tests on dry samples up to 90%. Deformations are based on samples containing cellulose waste and starch (K) as well as cellulose waste (P) and starch and hemp fibers (KA).

Type of Samples	Sample Acronym	The Maximum Deformation of Dry Samples	Stress σ_0_, Pa	Stress σ, Pa	Strain ε_0_, %	Strain ε, %	Calculated Young’s Modulus E, kPa
Control samples	K0.1-P1	90%	386	568	9.22	13.68	4.081
	K0.2-P2		2333	4595	4.90	7.08	103.761
	K0.3-P3		6158	10,897	3.69	5.19	315.933
	K0.4-P4		9454	17,657	6.83	9.20	346.118
	K0.5-P5		16,334	28,050	6.78	8.03	937.280
Samples with hemp	K0.05-KA0.05-P1		1082	2742	11.32	17.40	27.303
	K0.1-KA0.1-P2		2213	4378	1.48	2.88	154.643
	K0.15-KA0.15-P3		6928	14,554	20.28	23.59	230.387
	K0.2-KA0.2-P4		14,818	24,513	23.32	26.22	334.310
	K0.25-KA0.25-P5		9959	21,891	7.44	10.11	446.891

**Table 2:** Compression tests on oil-sorbed samples up to 70%. Deformations are based on samples with cellulose waste and starch (K) as well as samples with cellulose waste (P) and starch and hemp (KA).

Type of Samples	Sample Acronym	The Maximum Deformation of Oil-Sorbed Samples	Stress σ_0_, Pa	Stress σ, Pa	Strain ε_0_, %	Strain ε, %	Calculated Young’s Modulus E, kPa
Control samples	K0.1-P1	70%	153	334	4.10	9.33	3.461
	K0.2-P2		745	2718	6.92	9.93	65.548
	K0.3-P3		1155	5220	8.74	11.04	176.739
	K0.4-P4		5220	11,186	7.89	9.31	420.141
	K0.5-P5		19,294	46,407	4.50	6.60	1291.095
Samples with hemp	K0.05-KA0.05-P1		697	2068	18.22	24.34	22.402
	K0.1-KA0.1-P2		1660	3271	5.31	7.20	85.238
	K0.15-KA0.15-P3		1058	1996	5.93	7.01	86.852
	K0.2-KA0.2-P4		1130	3392	1.27	3.50	101.480
	K0.25-KA0.25-P5		4667	13,231	2.05	4.72	320.269

**Table 3:** Amounts of material used to prepare aerogels with starch [14].

Starch in g	Water in mL	Cardboard in g	Water in mL
0.1	20	1	80
0.2		2	
0.3		3	
0.4		4	
0.5		5	

**Table 4:** Material quantities of starch, hemp fibers, and cardboard for the control samples per 100 mL batch size [14].

Starch in g	Hemp Fibers in g	Water in mL	Cardboard in g	Water in mL
0.05	0.05	20	1	80
0.1	0.1	20	2	80
0.15	0.15	20	3	80
0.2	0.2	20	4	80
0.25	0.25	20	5	80

**Table 5:** Dry samples for mechanical measurements [14].

Type of Samples	Sample Acronym	Density, g cm^−3^	STDEV of Density, g cm^−3^	Porosity, %	STDEV of Porosity, %
Control samples	K0.1-P1	0.017	0.001	98.721	0.074
	K0.2-P2	0.027	0.002	96.050	0.302
	K0.3-P3	0.048	0.002	89.338	0.492
	K0.4-P4	0.057	0.003	83.337	1.017
	K0.5-P5	0.076	0.008	71.886	2.808
Samples with hemp	K0.05-KA0.05-P1	0.020	0.007	98.348	0.515
	K0.1-KA0.1-P2	0.030	0.002	95.426	0.306
	K0.15-KA0.15-P3	0.047	0.009	89.202	2.145
	K0.2-KA0.2-P4	0.060	0.004	81.927	1.300
	K0.25-KA0.25-P5	0.075	0.005	71.637	1.755

**Table 6:** Samples impregnated with MTMS for mechanical measurements [14].

Type of Samples	Sample Acronym	Density, g cm^−3^	STDEV of Density, g cm^−3^	Porosity, %	STDEV of Porosity, %
Control samples	K0.1-P1	0.018	0.003	98.687	0.187
	K0.2-P2	0.029	0.003	95.719	0.462
	K0.3-P3	0.042	0.005	90.643	1.062
	K0.4-P4	0.056	0.006	83.519	1.776
	K0.5-P5	0.075	0.009	72.495	3.161
Samples with hemp	K0.05-KA0.05-P1	0.023	0.001	98.255	0.077
	K0.1-KA0.1-P2	0.031	0.003	95.293	0.403
	K0.15-KA0.15-P3	0.051	0.005	88.295	1.136
	K0.2-KA0.2-P4	0.057	0.002	82.827	0.739
	K0.25-KA0.25-P5	0.068	0.004	74.357	1.544

## Data Availability

All data generated or analyzed during this study are included in this published article.
